# SPARQLGraph: a web-based platform for graphically querying biological Semantic Web databases

**DOI:** 10.1186/1471-2105-15-279

**Published:** 2014-08-15

**Authors:** Dominik Schweiger, Zlatko Trajanoski, Stephan Pabinger

**Affiliations:** Division for Bioinformatics, Biocenter, Innsbruck Medical University, Innsbruck, Austria; AIT – Austrian Institute of Technology, Health & Environment Department, Molecular Diagnostics, Vienna, Austria

**Keywords:** Semantic database, SPARQL, Semantic web, RDF, Database queries, Graphical query builder

## Abstract

**Background:**

Semantic Web has established itself as a framework for using and sharing data across applications and database boundaries. Here, we present a web-based platform for querying biological Semantic Web databases in a graphical way.

**Results:**

SPARQLGraph offers an intuitive drag & drop query builder, which converts the visual graph into a query and executes it on a public endpoint. The tool integrates several publicly available Semantic Web databases, including the databases of the just recently released EBI RDF platform. Furthermore, it provides several predefined template queries for answering biological questions. Users can easily create and save new query graphs, which can also be shared with other researchers.

**Conclusions:**

This new graphical way of creating queries for biological Semantic Web databases considerably facilitates usability as it removes the requirement of knowing specific query languages and database structures. The system is freely available at http://sparqlgraph.i-med.ac.at.

## Background

Nowadays, a plethora of biological data is freely available for the life sciences community. The vast majority of this data is accessible through heterogeneous relational databases and traditional keyword searching. As information is scattered across several databases using different data representations and formats, text based keyword searching and browsing often proves to be impractical. Effective research in life sciences is thereby currently hampered by the absence of integrated databases, and will get even more difficult as more and more biological data accumulates.

Over the last few years, the Semantic Web has established itself as a common framework allowing data to be used and shared across applications and database boundaries. Several biological Semantic Web databases and services for querying and integrating heterogeneous biological databases have emerged, trying to bring the advantages of Semantic Web to the life sciences community [1]. The Bio2RDF [2] project has converted and interconnected many biological databases, which allows creating queries across database boundaries. EBI very recently launched its own Semantic Web platform [3] for several of its databases, including UniProt, ChEMBL, and Reactome.

Together, these resources combine an enormous amount of biological information where typically profound background knowledge of the underlying databases as well as the query language is needed to access the information. SPARQL has emerged as the most-widely used query language to retrieve and manipulate data stored in Semantic Web databases, but often proves to be too complex for inexperienced users. Therefore, the task of querying the data remains an unresolved problem for many researchers. As a consequence, several efforts have been made to make the data more accessible and hide the complexities of the querying language from the end-user [4–8].

To the best of our knowledge, no service has been published that allows users to graphically build and execute biological Semantic Web queries without having to deal with the database schemas and underlying Semantic Web technologies. Therefore, we created the web-based platform SPARQLGraph featuring an intuitive graphical query editor, several predefined template queries, and a clear result presentation. The tool is open-source and freely accessible at http://sparqlgraph.i-med.ac.at.

## Implementation

The web application SPARQLGraph is based on JavaScript and uses the application framework Meteor [9] as backbone for both the client and server side. Meteor features rapid prototyping, and offers routing support, easy connections to a database, and HTML templating. SPARQLGraph uses the JavaScript graph visualization library mxGraph [10] for building and rendering graphs. This library allows designing of components, which mimic the behaviour of standalone applications in terms of functionality and design. Users of SPARQLGraph are authenticated and authorized by using the accounting system of Meteor. Furthermore, the commenting system DISQUS [11] is attached to each graph to enable specific discussions amongst users. SPARQLGraph currently supports Firefox, and Chrome and can be freely tested using a provided demo account.

## Results and discussion

### SPARQLGraph

SPARQLGraph is a web-based platform allowing users to build Semantic Web database queries in a novel, graphical way. The main interface of the platform consists of a large drawing board that is used to assemble new query graphs. Users can add new elements and their attributes to a query by simply dragging and dropping them onto the board.

**Figure 1:**
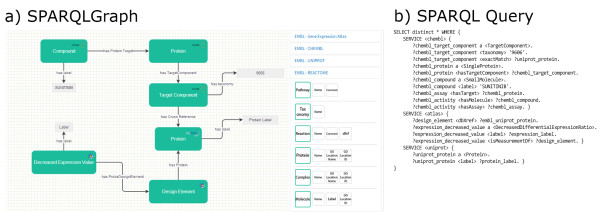
**Graphical and textual representation of an example query. a)** Displayed are the drawing board (left) and the element selection panel (right). Elements (objects and subjects) of the query are represented as green boxes that display the corresponding database in the top right corner. Relations between elements are represented as arrows and display the proper description. Properties of elements are represented as grey boxes. The depicted graph represents a federated query including three different databases (see example use case); **b)** shows the corresponding SPARQL query that is automatically generated by the system and executed at an endpoint.

Currently, SPARQLGraph supports several databases from the EBI RDF platform [3] and from the Bio2RDF project [2], which are listed in Table 1.Several core elements of the theses RDF databases were added to the system and important attributes were integrated into the application (see Figure 1, grey nodes). These attributes are specific to each element and can be used for filtering or extending query results. If possible, elements were cross linked between databases, which allows querying multiple databases at once using federated queries.

**Table 1:** **List of RDF databases integrated in SPARQLGraph**

EBI RDF platform				
Database	Triples	Focus	Example elements	Reference
Atlas	447.149.547	Gene Expression	Experiment, Assay	[12]
ChEMBL	374.762.364	Chemogenomics	Compound, Target	[13]
Reactome	12.487.422	Pathways	Pathway, Reaction	[14]
UniProt	9.024.662.088	Proteins	Protein	[15]
**Bio2RDF v2**				
**Database**	**Triples**	**Focus**	**Example Elements**	**Reference**
Entrez Gene	394.026.267	Genes	Gene	[16]
DrugBank	1.121.468	Drugs	Drug, Target	[17]
KEGG	49.850.774	Pathways	Pathway, Reaction	[18]
PharmGKB	142.782.063	Pharmacogenomics	Drug, Disease	[19]

Each database is listed with their number of triples, the area it focuses on, example database elements, and its reference.

In order to make the right connections between the selected elements, the platform verifies for allowed combinations and provides visual feedback to the user. Upon execution, the graph is automatically converted into the corresponding SPARQL query and submitted to an endpoint. The result is then displayed in a tabular form, which can also be exported in CSV format.

**Figure 2:**
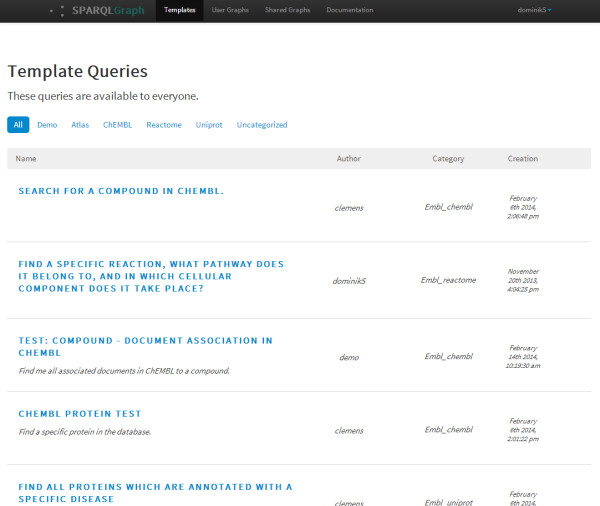
**Overview of example template queries.** Shown is a list of template queries in SPARQLGraph. Users can create new template queries or use them to quickly get answers to various biological questions.

Besides querying single databases, SPARQLGraph is also capable of creating federated queries where different databases are searched at the same time without the need for data transformation or manual result filtering.SPARQLGraph provides several manually curated template queries targeting different databases and use cases (see Figure 2). Template queries cannot be edited but allow substituting values of specific fields, for example gene name, protein name, or organism name. Therefore, they are of great help for answering common biological questions and for making new users familiar with the platform.

Every user has the possibility to create an account, where private queries can be stored. Moreover, template queries and private queries can be shared with the community to make them publicly accessible. The integrated commenting system allows users to quickly engage in discussions, suggest graph improvements, or propose query modifications. New users are guided through the system by a tutorial and are assisted during graph creation by tooltips and legends.

### Example use case

To illustrate the basic design of SPARQLGraph, we have selected the following example query: “Which protein targets of the sunitinib drug are down-regulated in any human tissue?”

Figure 1a) displays the graphical representation of the example query in SPARQLGraph, which is also available as a template query. The created federated query involves three different databases including ChEMBL (protein targets), Uniprot (protein), and Atlas (gene expression values). It starts with selecting the compound “SUNITINB” in the ChEMBL database. Next the compound is connected with all associated proteins and, in order to restrict it to human, it is linked with a target component having taxonomy 9606 (homo sapiens). To output the UniProt protein label of all associated proteins, a cross-reference between the ChEMBL database and the UniProt database is inserted. As gene expression patterns are stored in the Atlas database, a link from UniProt to Atlas is included. The Atlas design element represents a probe, which is used in an assay for detecting sequence or gene expression levels. To output only down-regulated proteins, the design element is connected to the *Decreased Expression Value* entity, which returns its label as a query result.Figure 1b) shows the generated SPARQL query, which is submitted to a corresponding endpoint. The query code is simplified for better readability.

## Discussion

Several approaches were made to facilitate the creation of SPARQL queries. Tools such as GRUFF [20], ViziQuer [21], and NITELIGHT [22] are using a very generic graph building approach often resulting in high complexity due to their comprehensive functionality. SPARQLGraph is using a different approach, in that it focuses solely on predefined biological databases, and puts emphasis on usability for users of the life science community. Furthermore, SPARQLGraph allows users to query several databases at once as it makes use of the SPARQL SERVICE keyword. This feature is crucial for using Semantic Web in the life science field, as more and more institutions offer their databases as linked RDF data [23] allowing for more comprehensive queries. To date, SPARQLGraph is the only query builder which integrates this valuable feature.

SPARQLGraph facilitates collaborative work amongst researchers by allowing them to create and share graphs. Therefore, users with similar biological questions can reuse and extend existing template queries to match their needs.

### Outlook

Future efforts on SPARQLGraph will focus on usability evaluation and simplification of the graphical query builder to further ease and speed up the query creation process. Furthermore, newly created RDF data sources will be linked to the currently supported ones, which would allow for more complex federated queries in SPARQLGraph. In order to support collaborations and the extension of the software we have submitted the code to a public repository on GitHub (https://github.com/tadKeys/sparqlgraph). In addition, the online user documentation provides an example of how to create new and extend existing database schemas within SPARQLGraph.

## Conclusions

We present SPARQLGraph, a web-based platform for the visual creation and execution of biological Semantic Web queries. The graphical query builder allows users to create and share query graphs in a new simple way. Several template queries are provided to offer a great starting point for building new graphs and assist researchers in finding answers to biological questions. Currently, the requirements of knowing the querying language SPARQL and the exact structure of the used databases are limiting the success of biological Semantic Web platforms. SPARQLGraph tries to remove these burdens from the user and considerably facilitates the creation of biological Semantic Web database queries. The platform actively supports user collaborations through an integrated commenting system and can be extended with additional databases.

## Availability and requirement

-  
**Project name:** SPARQLGraph
-  
**Project home page:**http://sparqlgraph.i-med.ac.at & https://github.com/tadKeys/sparqlgraph
-  
**Operating system(s):** Platform independent
-  
**Programming language:** JavaScript
-  
**Other requirements:** Modern Browser, i.e. current version of Firefox or Chrome
-  
**License:** GNU GPL
-  
**Any restrictions to use by non-academics:** mxGraph is available under the non-commercial Creative Commons
